# Temperature Dependence of Mechanical, Electrical Properties and Crystal Structure of Polyethylene Blends for Cable Insulation

**DOI:** 10.3390/ma11101922

**Published:** 2018-10-09

**Authors:** Lunzhi Li, Lisheng Zhong, Kai Zhang, Jinghui Gao, Man Xu

**Affiliations:** State Key Laboratory of Electrical Insulation and Power Equipment, Xi’an Jiaotong University, Xi’an 710049, China; demoniacpea@stu.xjtu.edu.cn (L.L.); zhangkai.925@stu.xjtu.edu.cn (K.Z.); xumman@mail.xjtu.edu.cn (M.X.)

**Keywords:** polyethylene blends, electrical properties, mechanical properties, crystal structure, cable insulation

## Abstract

There is a long-standing puzzle concerning whether polyethylene blends are a suitable substitution for cable-insulation-used crosslinking polyethylene (XLPE) especially at elevated temperatures. In this paper, we investigate temperature dependence of mechanical, electrical properties of blends with 70 wt % linear low density polyethylene (LLDPE) and 30 wt % high density polyethylene (HDPE) (abbreviated as 70 L-30 H). Our results show that the dielectric loss of 70 L-30 H is about an order of magnitude lower than XLPE, and the AC breakdown strength is 22% higher than XLPE at 90 °C. Moreover, the dynamic mechanical thermal analysis (DMA) measurement and hot set tests suggest that the blends shows optimal mechanical properties especially at high temperature with considerable temperature stability. Further scanning electron microscope (SEM) observation and X-ray diffraction (XRD) analysis uncover the reason for the excellent high temperature performance and temperature stability, which can be ascribed to the uniform fine-spherulite structure in 70 L-30 H blends with high crystallinity sustaining at high temperature. Therefore, our findings may enable the potential application of the blends as cable insulation material with higher thermal-endurance ability.

## 1. Introduction

The extruded cable employs XLPE (crosslinking polyethylene) as its insulation material, owing to its preferable electrical and mechanical performance. Nevertheless, such a material suffers from its environmental impact, because there is a high level of greenhouse gas emission during its manufacture, and a low level of recyclability at the end of its lifetime due to chemical crosslinking. Therefore, developing new recyclable polyethylene insulating materials substituting the conventional XLPE has been becoming a long-standing topic.

Polymer blending has been proved to be an effective and noteworthy approach for the material modification for a variety of properties [1,2,3,4]. Thermoplastic polymer blends are considered as a new type of environmental-friendly cable insulation material substituting XLPE, which exhibits lower energy consumption in the production process and better recyclability at the end of service life [5,6,7]. This has triggered increasing research interests in the past two decades. Hosier et al. have performed a series of work on polyethylene (PE) blends. It was found that the blends of 80 wt % low density polyethylene (LDPE) and 20 wt % HDPE can produce space filling texture spherulite and reduce the concentration of impurities and defects, leading to an improvement in the electrical strength and mechanical properties [8,9,10]. Moreover, the mini-cables are manufactured and show higher DC breakdown strength than XLPE mini-cables [11]. Besides, lots of research has been conducted on polypropylene (PP) based blends. The addition of polyolefin elastomer (POE) to PP can improve mechanical flexibility at room temperature [12]. Propylene-ethylene copolymer (PEC)/ isotactic polypropylene (iPP) blends can enhance flexibility impact resistance at low temperature, and suppress space charge accumulation [13]. Furthermore, the DC tests for mini cables with PP blending material show higher electrical strength than that of XLPE [14]. Our earlier investigations report the modification of LLDPE as matrix resin by blending with HDPE. It is found that the mechanical and electrical performance of blends containing 70% wt LLDPE and 30% wt HDPE could be obviously improved and could be even better than XLPE [15,16,17,18]. Further studies indicate that the electrical aging resistance was also promoted for optimized blends [19].

However, the increasing voltage level and capacity imposes stringent requirement on the cable insulation materials. For example, the cable system (including insulation material) always works at an elevated temperature. Therefore, the temperature dependence of properties for blends is of great significance for their applications in cable insulation. In this paper, the mechanical properties and electrical properties of blends were studied with the change of temperature, as well as the morphology and the crystal structure of blends. The variation of its structure with temperature and its influence mechanism on mechanical and electrical properties are also discussed.

## 2. Materials and Methods

### 2.1. Sample Preparation

The matrix resins used for blending are commercial LLDPE (0.923 g/cm^3^ in density and 0.25 g/10 min in melt index) and HDPE (0.945 g/cm^3^ in density and 0.75 g/10 min in melt index). The blends containing 70% wt LLDPE and 30% wt HDPE with antioxidant were mixed by a twin screw extruder at 180 °C which is followed by granulation after cooling. The granules were further melt pressed at 175 °C, 10MPa for 10 min into different thicknesses required for the corresponding tests. For comparison groups, LLDPE fabricated was made by using the same melt pressing procedure as the blends, and the XLPE was a commercialized product for 110 kV cable insulation.

### 2.2. Electrical Properties Tests

The dielectric constant, dielectric loss, and electrical breakdown strength are carried out at 30 °C, 45 °C, 60 °C, 75 °C and 90 °C respectively. The specimens are 100 × 100 × 10 mm for dielectric constant and loss factor tests by using the high voltage Schering bridge (Haefely, Basel, Switzerland) with the 50 Hz, 1 kV as test voltage. The sample thickness for breakdown tests is about 100 μm. In breakdown tests, a spherical-cylindrical electrode is used and the samples and electrodes are immersed in vegetable insulation oil to prevent surface flashover and swelling. A 50 Hz alternating (AC) voltage is applied to the sample with a root mean square (RMS) amplitude ramp of 1.5 kV/S until breakdown occur.

### 2.3. Mechanical Properties Tests

The variation of mechanical modulus with a temperature range (25–140 °C) is measured by dynamic mechanical thermal analysis (DMA, Mettler Toledo, Zurich, Switzerland). The samples fixed by dual cantilever beam fixture are 50 × 10 × 5 mm and are tested under the sinusoidal force of 1 N and 1 Hz.

The creep behavior of high temperature is characterized by hot set tests. The standard dumbbell type sample is adopted with the testing load of 20 N/cm^2^ under testing temperature range from 90–140 °C with an interval of 10 °C and testing time of 60 min.

### 2.4. Morphological Structure Characterization

The specimens used for SEM observation are etched in 95 wt % concentrated sulfuric acid/ 5 wt % potassium permanganate solution for 1 h, then rinsed by ultrasonic oscillator and treated by gold-sputtering successively. The structural parameters of materials at different temperatures are characterized by XRD. The scanning range of 2θ is 10°–30° with a scanning rate of 9.6°/min.

## 3. Results

### 3.1. Temperature Dependence of Electrical Properties

**Figure 1:**
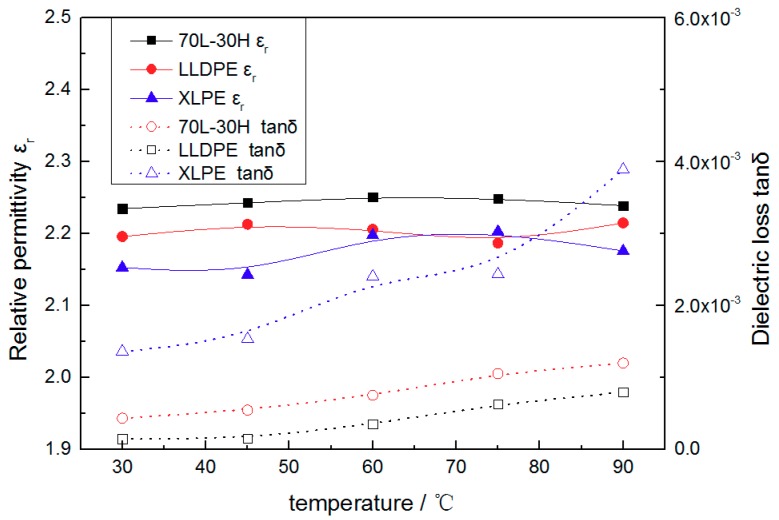
The relative permittivity and dielectric loss of 70 L-30 H, LLDPE and XLPE with temperature.

The relative permittivity (ε_r_) and dielectric loss (tan*δ*) of 70 L-30 H blends, LLDPE and XLPE with temperature are shown in Figure 1. It can be seen that the relative permittivity of three materials are very close with no obvious change trend with the temperature. This is because polyethylene is a typical non-polar organic material, whose polarization is mainly electronic displacement polarization with no turning-direction polarization. The electronic displacement polarization does not change with temperature; thus the permittivity of polyethylene material will not vary with temperature [20]. The blends have low permittivity similar to XLPE, which can meet the requirements of insulation materials for cables.

The dielectric loss of blends is about an order of magnitude lower than XLPE at room temperature and the growth rate of blends is obviously smaller than that of 70 L-30 H with the increase of temperature. The dielectric loss of polymer mainly includes conductive loss and dielectric polarization loss. As a typical non-polar polymer, PE has no turning polarization loss, so its dielectric loss is mainly conductivity loss. The mechanical properties of the material have little effect on the conductivity, whereas the number and mobility of carriers are the main factors [20]. The blends show the lowest loss factor, which means they have the lowest volume conductivity. Lower loss factor leads to lower line loss and heating, so it is vital for reducing line losses and limiting the temperature rising in cable system. There is significant difference in dielectric loss between XLPE and 70 L-30 H blends, indicating large difference between XLPE and blends in the number and mobility of carriers.

**Figure 2:**
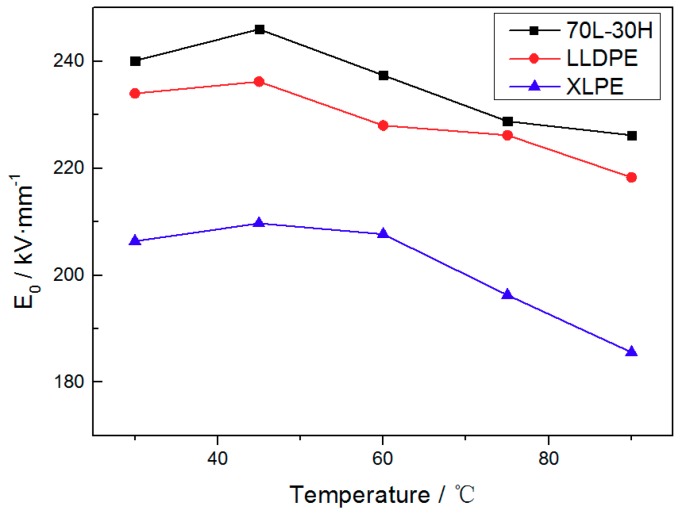
The *E*_0_ of 70 L-30 H, linear low density polyethylene (LLDPE) and crosslinking polyethylene (XLPE) with temperature.

The breakdown of insulation materials obeys the two parameters Weibull distribution. According to the test results, the scale parameter *E*_0_ (indicates the electric field strength at breakdown probability of 63.2%, which is used to characterize the breakdown strength of materials) of three materials at different temperatures can be calculated by mathematical software (Minitab 16) and shown in Figure 2. It can be seen from the figure that the breakdown strength of three materials first increase and then decrease with temperature. The blends have the highest breakdown strength at high temperature, and is significantly higher (about 22%) than XLPE at 90 °C, exhibiting better electrical strength and temperature stability at high temperature.

### 3.2. Temperature Dependence of Mechanical Properties

**Figure 3:**
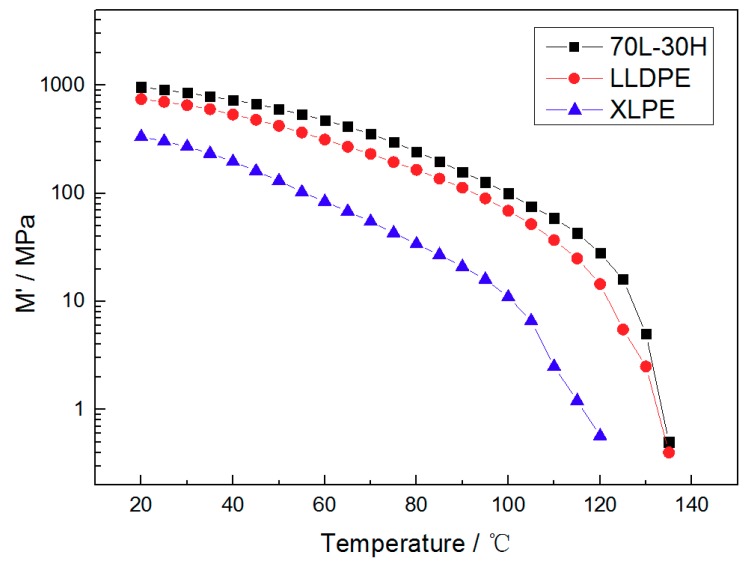
The temperature spectrum for dynamic thermo mechanical of 70 L-30 H, LLDPE and XLPE.

Figure 3 shows the variation of mechanical modulus with temperature for blends and LLDPE, XLPE. The mechanical modulus of the three materials decrease with the temperature increasing, because the thermal motion of polyethylene molecules increases with the temperature rising, and the material became soft gradually. It can be found from the figure that the blends can maintain the highest mechanical modulus at the same temperature compared to LLDPE and XLPE, exhibiting excellent high temperature mechanical properties in the tested temperature range. Table 1 shows the mechanical modulus of different materials at 30 °C and 120 °C, and analyzes the temperature dependence of the mechanical properties by using the ratio of mechanical modulus at 120 °C and 30 °C. It can be seen that the mechanical modulus of blends is about two times that of LLDPE and nearly 60 times that of XLPE at 120 °C. The ratio of blends is also the highest, which shows the best temperature stability. Such enhancement and temperature stability of blends in mechanical modulus will be of great importance at high temperature in a real cable system [11].

**Figure 4:**
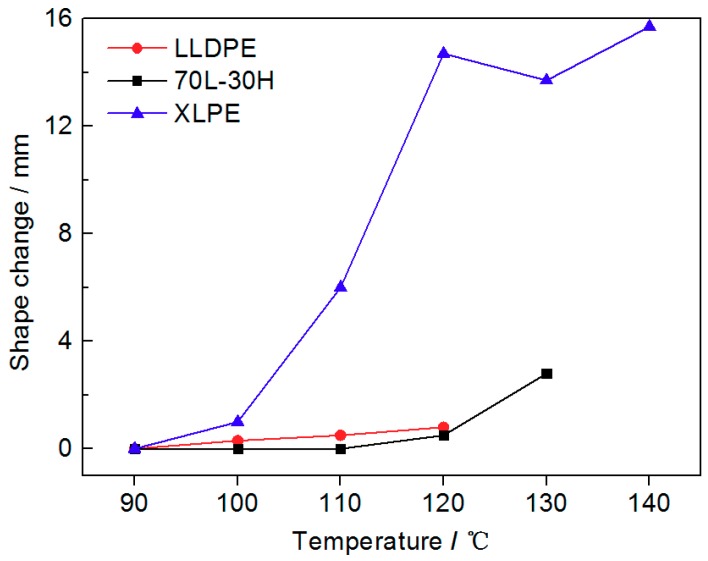
Test results of hot set tests with temperature.

Figure 4 shows the results of hot set tests of three materials at different temperatures. The initial deformation temperature of LLDPE and XLPE is 100 °C, while this temperature of 70 L-30 H blends rises about 20 °C to 120 °C. The abruption temperature of LLDPE is 130 °C, while that of blends rises 10 °C to 140 °C. It is worth noting that the 70 L-30 H blends achieve this considerable promotion with the melting point rises only one degree than neat LLDPE (from our previous differential scanning calorimetry (DSC) results) [16]. The deformation of XLPE increases rapidly with the increase in temperature at first, but only changes slightly when the temperature is over 120 °C. The 70 L-30 H blends shows small deformation with only 2.8 mm elongation at 130 °C after 60 min, owing to its higher modulus at high temperature from DMA results.

### 3.3. Structure Characterization

**Figure 5:**
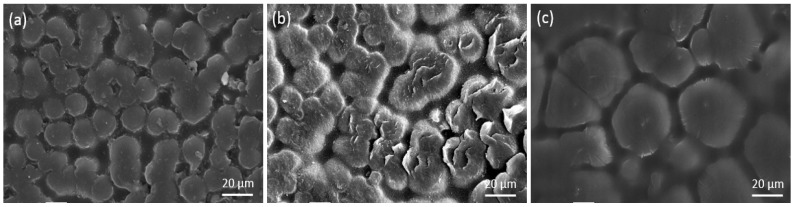
SEM micrographs showing samples of different materials after etching: (**a**) 70 L-30 H, (**b**) LLDPE, and (**c**) XLPE.

Figure 5 shows the SEM morphology of 70 L-30 H blends (a), LLDPE (b) and XLPE (c) with 1000 times magnification. The three materials show different spherulite structure obviously. The blends exhibit a more uniform and regular arrangement compared to LLDPE with similar spherulite size (about 10 μm in diameter), which contributes to HDPE molecules with high linearity acting as homogeneous nucleation. Such uniform fine-spherulite structure can suppress the concentration of amorphous regions and impurities. XLPE shows the largest spherulite size (about 20 μm in diameter) which is about two times than that of the blends, leading to the concentration of amorphous regions and impurities.

**Figure 6:**
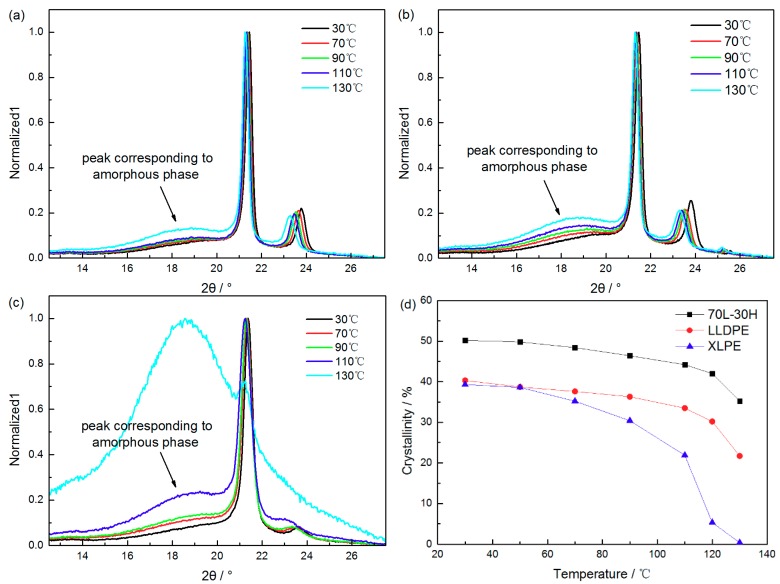
The XRD scanning spectrum for 70 L-30 H (**a**), LLDPE (**b**), XLPE (**c**) and the variation of crystallinity with temperature (**d**).

Figure 6 shows XRD patterns of 70 L-30 H (a), LLDPE (b), and XLPE (c) at different temperatures. According to calculating results, the diffraction error caused by thermal expansion is negligible. It can be seen that all of three materials show the characteristic crystallization peaks (110) and (200) of polyethylene. With the increase of temperature, the characteristic peaks shift to small degree slightly, and the area corresponding to the amorphous phase (18°–19°) increases gradually. XLPE shows no obvious crystallization peak at 130 °C, indicating that XLPE has completely melted at this temperature. According to the normalized XRD patterns, the crystallinity of three materials can be calculated and shown in Figure 6d.

It can be seen from the figure that the crystallinity of blends is the highest, while XLPE is the lowest. With the increase of temperature, the crystallinity of the three materials show downward trends. The crystallinity of XLPE preforms the fastest downward trend, nearly to 0 at 130 °C. The crystallinity of blends maintains the highest crystallinity at 130 °C (almost two times than that of LLDPE), with the melting point is only raised by 1 °C than LLDPE. The obvious enhancement of crystal structure at this high temperature may improve the temperature stability of its properties. The detailed discussions will be given in following section.

## 4. Discussion

The manipulation of grain size plays an important role on the material performance of polymer blending. In this paper, due to its molecules with high linearity and high melting point, HDPE is added into LLDPE matrix resin to modify the crystal structure of the blends. In the crystallization process of LLDPE/HDPE blends, HDPE can act as the homogeneous nucleus and raise the crystallizing temperature of blends. The increasing in the number of nucleus will promote the formation of small spherulites. Meanwhile, the accompanied nucleation of HDPE can drive more molecules to crystallize and elevate the overall crystallinity of the blends. Therefore, the blends can maintain relatively stable structure and higher crystallinity at even higher temperatures, resulting in a better temperature stability.

Temperature plays an important role in aggregation state of polyethylene, and the aggregation state greatly influences on the mechanical and electrical properties of materials. The following contents are discussed from two aspects: i) electrical and ii) mechanical.

(i) The loss factor of blends and XLPE is obviously different under 50 Hz AC electric field. According to the dielectric behavior of the polyethylene, the loss is basically the conductive loss, which illustrates that blends shows a lowest volume conductivity. The conductivity of polyethylene under low electric field depends on the number and mobility of impurity ions carriers. On one hand, the non-crosslinking blending process avoids the introducing of crosslinking agent, which limits the number of impurity carriers compared with XLPE. On the other hand, the blends show a uniform fine-spherulite structure. Such structure limits the mobility of impurity carriers [21]. Whereas, the big spherulite structure of XLPE can concentrate in the amorphous region, causing the increase in mobility. Thus, the conductive loss of blends is the lower than XLPE. Furthermore, the blends can maintain high crystallinity as the temperature rises, and restrict the enlargement of amorphous region as well as mobility of impurity carriers, leading to optimal temperature stability of loss factor.

The electrical strength of polyethylene materials is related to its morphological structure and mechanical properties [20,22,23]. The breakdown strength of three materials increased slightly with the increase of temperature below 45 °C. This is because the structure of material does not change significantly in this temperature range, but the lattice vibration increases with the temperature rising, increasing the collision number between electrons and lattice, reducing the free path of the electrons. According to the electromechanical breakdown theory, when temperature continues to rise, the decline of breakdown strength is mainly contributed to the decreasing of mechanical modulus [23,24,25,26]. The blends can form uniform fine-spherulite structure to increase electron scattering [27,28], and can maintain high crystallinity and high mechanical modulus at elevated temperature, leading to an enhanced breakdown strength at high temperature and the best temperature stability.

(ii) In General, the mechanical behaviors of polymers are dependent upon both crystalline phases and their interactions in amorphous regions [29,30]. Compared with neat LLDPE and XLPE, the uniform fine-spherulite structure of blends leads to closer physical connection between spherulite. Besides, it is found that the addition of HDPE in 70 L-30 H can increase lamellae thickness and promote the number of thick lamellae to perfect the crystal, form a thick lamellae filling configuration in amorphous regions to make more uniform phase structure according to our previous work [19]. Therefore, both the crystalline phases and physical interactions between crystalline phases have been strengthened, leading to enhanced mechanical properties for blends. When the temperature rises, the HDPE molecules which act as nucleation and thick lamellae show a higher melting temperature than XLPE, so they still play the role of sustaining crystal structure and mechanical stress transfer units, exhibiting the best temperature stability for mechanical modules. Also, it is a convincing explanation for that the initial deformation temperature and the maximum tolerance temperature of blends are 20 °C and 10 °C higher than LLDPE under load, with only 1 °C higher melting point.

The reasons for the excellent electrical and mechanical properties of the blends were analyzed by morphological structure. It was found that the properties of the blends above 100 °C are comparable to those of XLPE at 90 °C. Therefore, the long-term service temperature of the 70 L-30 H blends may exceed 100 °C, which is of great value for the actual operation cable system.

## 5. Conclusions

In conclusion, the optimal electrical properties with a loss factor one magnitude lower than XLPE and an electrical strength 22% higher than XLPE at 90 °C can be found in 70 L-30 H blends. From the dynamic mechanical thermal analysis and hot set tests, it is also found that blends show outstanding mechanical modules and creep behaviors especially at high temperature. In the temperature range of 30–130 °C, the deterioration of blends decreases slower than XLPE showing better temperature stability. Morphological structure obtained by SEM observation and XRD analysis suggests that the 70 L-30 H blends forms a uniform fine-spherulite structure with high crystallinity at high temperature. Such structure characteristic convinces the excellent performance at high temperature and considerable temperature stability of blends.

## Figures and Tables

**Table 1:** Mechanical modulus of specific temperature points and their ratio.

Materials	*M*’(30 °C)/MPa	*M*’(120 °C)/MPa	*M*’(120 °C)/*M*’(20 °C)
70 L-30 H blends	857	33	0.038
LLDPE	655	14.5	0.022
XLPE	221	0.57	0.0025
